# Supplementary material for: Pen-grip kinetics in children with and without handwriting difficulties
Source: PLoS One. 2022 Jun 24;17(6):e0270466. doi: 10.1371/journal.pone.0270466 (PMC9231762; doi:10.1371/journal.pone.0270466)
Supplement: S1 Table — (DOCX) [file pone.0270466.s001.docx]

S1 Table. Summary Table of the Parameters for the Three Subtypes of HD Group.

| Kinetics of handwriting  (sources of force) | HD subtype | | | | | |
| --- | --- | --- | --- | --- | --- | --- |
|  | Cognitive Learning Dysfunction  (n=7) | | Motor Impairment  (n=8) | | Severe Hybrid  (n=13) | |
|  | Mean | SD | Mean | SD | Mean | SD |
| Fine motor skills | | | | | | |
| Fine motor precision | 33.85 | 3.48 | 34.75 | 2.65 | 31.61 | 6.35 |
| Fine motor integration | 33.28 | 3.98 | 35.87 | 3.27 | 32.92 | 7.36 |
| Manual dexterity | 26.28 | 3.90 | 26.12 | 3.27 | 25.31 | 6.44 |
|  |  |  |  |  |  |  |
| Average Force (AF), unit: Newton | | | | | |  |
| Thumb -on paper | 2.95 | 0.22 | 2.89 | 0.52 | 2.41 | 0.70 |
| Thumb -in air | 1.11 | 0.24 | 1.11 | 0.29 | 1.12 | 0.48 |
| Thumb -whole task | 2.31 | 0.30 | 2.45 | 0.43 | 2.11 | 0.66 |
|  |  |  |  |  |  |  |
| Index -on paper | 1.98 | 0.84 | 1.79 | 0.67 | 1.60 | 0.45 |
| Index -in air | 0.62 | 0.17 | 0.61 | 0.21 | 0.71 | 0.32 |
| Index -whole task | 1.67 | 0.71 | 1.49 | 0.55 | 1.42 | 0.41 |
|  |  |  |  |  |  |  |
| Middle -on paper | 1.41 | 0.88 | 0.94 | 0.38 | 1.01 | 0.39 |
| Middle -in air | 0.85 | 0.45 | 0.67 | 0.21 | 0.74 | 0.29 |
| Middle -whole task | 1.31 | 0.88 | 0.90 | 0.34 | 1.04 | 0.35 |
|  |  |  |  |  |  |  |
| Pen-Tip -on paper | 0.88 | 0.41 | 0.83 | 0.41 | 0.66 | 0.29 |
|  |  |  |  |  |  |  |
| Task Time (TT) | | | | | |  |
| TT -on paper (ratio) | 0.71 | 0.04 | 0.73 | 0.04 | 0.71 | 0.06 |
| TT -in air (ratio) | 0.28 | 0.04 | 0.26 | 0.04 | 0.29 | 0.06 |
| TT -whole task (s) | 24.01 | 7.35 | 31.02 | 11.71 | 26.38 | 10.18 |
|  |  |  |  |  |  |  |
| Coefficient of Variation in Force (CVF) | | | | | |  |
| Thumb -whole task | 0.39 | 0.08 | 0.43 | 0.07 | 0.36 | 0.07 |
| Index -whole task | 0.47 | 0.11 | 0.55 | 0.21 | 0.43 | 0.10 |
| Middle -whole task | 0.46 | 0.12 | 0.47 | 0.06 | 0.37 | 0.12 |
| Pen-Tip -on paper | 0.37 | 0.05 | 0.36 | 0.07 | 0.31 | 0.04 |
|  |  |  |  |  |  |  |
| Number of Force Fluctuations per second (NFFPS) | | | | | |  |
| Thumb -whole task | 1.29 | 0.15 | 1.15 | 0.11 | 1.31 | 0.18 |
| Index -whole task | 1.34 | 0.19 | 1.15 | 0.11 | 1.28 | 0.26 |
| Middle -whole task | 1.47 | 0.26 | 1.25 | 0.17 | 1.44 | 0.26 |
|  | 0.11 |  |  |  |  |  |
| Force Ratio (FR) -on paper | 0.11 | 0.10 | 0.12 | 0.03 | 0.11 | 0.03 |
| *Note.* SD = standard deviation; HD = handwriting difficulty | | | | | | |
