# Supplementary material for: Pen-grip kinetics in children with and without handwriting difficulties
Source: PLoS One. 2022 Jun 24;17(6):e0270466. doi: 10.1371/journal.pone.0270466 (PMC9231762; doi:10.1371/journal.pone.0270466)
Supplement: Attachment — Submitted filename: PONE-D-21-12263 - Review.pdf [file pone.0270466.s002.pdf]

PONE

PONE-D-21-12263

REVIEW

The article entitled “Pen-grip Kinetics in Children with and without Handwriting Difficulties” describes an experimental research. Children with and without handwriting difficulties (HD) were evaluated by means a pen with force sensors while writing numbers in a previously printed in grey sheet without any other constraints. The force applied by the three fingers – thumb, index and middle – was recorded along the period when the pen was in touch with the sheet and in the air.

The Introduction Section was well designed, and the lecture easily flows toward the objectives presented at its end. However, only one article cited in this section was published in the last five years and nine from the 23 of them were published in the last century. This selection makes us wonder the relevance of the research object and the addition that the results to be obtained can bring to what is already described in the literature.

This wondering rises when the sample is detailed. Three HD forms are found in the sample: “a cognitive learning dysfunction subtype (25%), a motor impairment subtype (28.6%), and a severe hybrid subtype (46.4%).” Further on, the text describes each of these types of HD: “The severe hybrid subtype is the most serious subtype, reflecting severe deficits in all five dimensions and poor perceptual motor ability” ... “The motor impairment subtype is mainly characterized with deficits in writing speed, construction of characters, DCD, writing automation, and fine motor control” … “Children classified into the cognitive learning dysfunction subtype lack appropriate, efficient learning strategies”. In this sense, the differences between each subtype are significant, either by severity or specificity. However, the sample is treated as a single group so that there can be statistical strength. Finally, at the beginning of the discussion, when commenting on the homogeneity of manual dexterity between the groups, it is stated that “this might be related to the fact that most of the writing difficulty subtypes were “motor impairment” and “severe hybrid,” which are both related to poor motor performance during handwriting”, which demonstrates an important reductionism of the type "severe hybrid". This statement also puts a quarter of the sample out of the discussion by assuming that the argument used for both of that types would not apply to the "cognitive learning dysfunction" type.

In addition, the study observed five different categories of handwriting grip with different intra-group and – mainly – between-groups distributions. There is no mention of the distribution of these categories of handwriting grip in the three types of HD in the sample.

There is an important phrase related to this topic whose wording is ambiguous in my opinion:

"Although the positions of the three force sensors on FAP are adjustable, in this study, all participants used the same position setting for the force sensors based on their own grip."

What does it mean? That all children used the same position setting of the sensors in the pen, regardless of how each child grasped it? Or that each child had an individualized configuration of sensors according to their form of grasping? Or, yet, that there were several configurations, one for each form of handwriting grasping, but not one for each child according to their natural positioning?

This questioning is important because it can indicate whether there was an extra constraint factor for the HD group. If the positioning was unique, its effects on the HD group may have been greater than on the group without HD. If the positioning was determined by the way of grasping, how many subgroups were formed? And if the positioning was individualized by a child, what did you mean by "same position setting"?

Therefore, I believe that, by not adequately considering the heterogeneity of the HD sample, the authors compromised the reliability of the results. As stated by them, "due to the small sample size and uneven percentages among the subtypes, differences between HD subtypes were not analyzed".

Some minor considerations:

The model of direct citations used in the article (citing only the first two authors) is not adequate, since it gives a single comma the power to indicate that there are other authors besides the two mentioned. Forms such as "Author and colleagues", "Author and collaborators" and even "Author et al." are preferable in these cases.

Although it could be considered as implied, the text does not formally indicate whether the sample is composed only by right-handed children and does not comment on the consistency of manuality. They belong to a key age range regarding manuality definition, and the topic deserves to be considered in the characterization of the sample (Scharoun & Bryden, 2014).

The Cronbach's alpha value for the BOT-2 test is 0.92; however, ideal values for Cronbach's alpha range from 0.7 to 0.9. Values greater than 0.9 indicate test redundancy (Streiner, 2003).

Right following the phrase “There have been few studies reporting on applied force while pen-lifting” there are no references to these few studies, nor a discussion comparing the results obtained in the study with this literature, which is very relevant here.

Scharoun, S. M. and Bryden, P. J. Hand preference, performance abilities, and hand selection in children. Front Psychol. 2014; 5: 82. doi: 10.3389/fpsyg.2014.00082

Streiner, D. L. Being inconsistent about consistency: when coefficient alpha does and doesn’t matter. Journal of Personality Assessment. 2003; 80:217-222. doi: 10.1207/S15327752JPA8003_01
