# Supplementary material for: Pen-grip kinetics in children with and without handwriting difficulties
Source: PLoS One. 2022 Jun 24;17(6):e0270466. doi: 10.1371/journal.pone.0270466 (PMC9231762; doi:10.1371/journal.pone.0270466)
Supplement: Attachment — Submitted filename: Response to Reviewers.docx [file pone.0270466.s003.docx]

Reviewer #1:

The article entitled “Pen-grip Kinetics in Children with and without Handwriting Difficulties” describes an experimental research. Children with and without handwriting difficulties (HD) were evaluated by means a pen with force sensors while writing numbers in a previously printed in grey sheet without any other constraints. The force applied by the three fingers – thumb, index and middle – was recorded along the period when the pen was in touch with the sheet and in the air.

We sincerely appreciate the Reviewer’s thorough review and valuable suggestions. The authors revised the manuscript according to your constructive comments. The point-by-point responses are shown as follows:

1. The Introduction Section was well designed, and the lecture easily flows toward the objectives presented at its end. However, only one article cited in this section was published in the last five years and nine from the 23 of them were published in the last century. This selection makes us wonder the relevance of the research object and the addition that the results to be obtained can bring to what is already described in the literature.

**Response:** Thanks for the constructive comments. The authors have searched and added more suitable published studies as references in the Introduction Section of the revised version of the paper. Please see the following lists of the additional articles cited in this revised manuscript. While searching for these updated citations, the authors also found that the research works related to children’s handwriting kinetics have become more scarce in recent years. Therefore, in addition to classic articles related to handwriting kinetics published several decades ago, most of the studies, as the reviewer mentioned, are works from the period 2005 to 2015, which was an excellent time period for handwriting researchers to discuss the pen tip force based on the mature writing tablet (Wacom) system. However, direct evidence regarding the finger/digit forces acting on the pen barrel is still scant, so our team (and other previous works cited in this manuscript) established a Digit Forces Acquisition pen that is similar to a real pen (either shape or weight/size). With technology that can measure small forces incorporating pressure sensors as well as the 3D printing technical advancements, we believe that handwriting kinetics-related studies may be conducted easily for further the purpose of discussing the mechanisms of handwriting motor or processing functions. Thanks again for the suggestion, which motived us to updated our citations in the revised Introduction section.

- Hochhauser M, Wagner M, Shvalb N. Assessment of children's writing features: A pilot method study of pen-grip kinetics and writing surface pressure. Assist Technol. 2021. Epub 2021/07/22. doi: 10.1080/10400435.2021.1956640.
- Lin Y-C, Chao Y-L, Hsu C-H, Hsu H-M, Chen P-T, Kuo L-C. The effect of task complexity on handwriting kinetics. Canadian Journal of Occupational Therapy. 2019;86(2):158-68. doi: 10.1177/0008417419832327
- Gatouillat A, Dumortier A, Perera S, Badr Y, Gehin C, Sejdic E. Analysis of the pen pressure and grip force signal during basic drawing tasks: The timing and speed changes impact drawing characteristics. Comput Biol Med. 2017;87:124-31. Epub 2017/06/06. doi: 10.1016/j.compbiomed.2017.05.020.

2. This wondering rises when the sample is detailed. Three HD forms are found in the sample: “a cognitive learning dysfunction subtype (25%), a motor impairment subtype (28.6%), and a severe hybrid subtype (46.4%).” Further on, the text describes each of these types of HD: “The severe hybrid subtype is the most serious subtype, reflecting severe deficits in all five dimensions and poor perceptual motor ability” ... “The motor impairment subtype is mainly characterized with deficits in writing speed, construction of characters, DCD, writing automation, and fine motor control” … “Children classified into the cognitive learning dysfunction subtype lack appropriate, efficient learning strategies”. In this sense, the differences between each subtype are significant, either by severity or specificity. However, the sample is treated as a single group so that there can be statistical strength.

**Response:** Thanks for the comments. Considering that the original and primary purpose of this study was to reveal the difference in pen grip kinetics in the handwriting performance of an HD group and a control group, the CHEF was used in this study mainly to verify that the children in the HD group indeed had handwriting problems. Distinguishing among the differences in the writing kinetics among the HD subtypes, however, was not the original purpose of this study. Nevertheless, the authors agree with the reviewer’s comment that the differences in the writing characteristics among each HD subtype might be significant or interesting topics to be explored further. Therefore, the authors have stated this in the Discussion section and the Limitations and Future Studies part of this revised version as “**However, the data in this study were analyzed in children with uneven percentages of distribution among all three subtypes of HD. Since HD is very complex, and an assessment for HD classification has only been developed recently, the results obtained in this study should be interpreted with caution when generalizing the findings to children with different types of HD**” and ” **Finally, although children with three subtypes of HD were reported according to the CHEF classification, this study did not analyze the differences of the characteristics of writing kinetics among the different HD subtypes due to the small sample size and uneven percentages of distribution among the subtypes in the current study sample. A future study with a larger sampling scale is suggested to compare the differences of the writing features among HD subtypes.”**

Please refer to Page 16 lines 262-266 and Page 21 lines 378-383, respectively.

3. Finally, at the beginning of the discussion, when commenting on the homogeneity of manual dexterity between the groups, it is stated that “this might be related to the fact that most of the writing difficulty subtypes were “motor impairment” and “severe hybrid,” which are both related to poor motor performance during handwriting”, which demonstrates an important reductionism of the type "severe hybrid". This statement also puts a quarter of the sample out of the discussion by assuming that the argument used for both of that types would not apply to the "cognitive learning dysfunction" type.
   **Response:** Thanks for the comments. The authors agree with the reviewer’s viewpoint. The original statement was misused to assume that the argument used for both of that types would not apply to the "cognitive learning dysfunction" type. The authors thus deleted the original sentences **“This might be related to …poor motor performance during handwriting.”** and added the following sentences: **“However, the data in this study were analyzed in children with uneven percentages of distribution among all three subtypes of HD. Since HD is very complex, and an assessment for HD classification has only been developed recently, the results obtained in this study should be interpreted with caution when generalizing the findings to children with different types of HD.”** at the end of this paragraph. As mentioned in the previous question (point 2), the characteristics of each HD subtype could be an important issue. Therefore, the authors have also stated this issue as one of the study limitations in the **Limitations and Future Studies** section.

Please refer to Page 16 lines 262-266 and Page 21 lines 375-376, respectively.

4. In addition, the study observed five different categories of handwriting grip with different intra-group and – mainly – between-groups distributions. There is no mention of the distribution of these categories of handwriting grip in the three types of HD in the sample.

**Response:** Thanks for the comments. There are two reasons why the authors did not mention the distribution of these handwriting grip categories in the three types of HD in our recruited participants. The first reason is that all the children recruited in this study could use the tripod grasp to write. This grip type was recorded as the main grip type when a child uses a regular or typical pencil to write because it is usually taught as the standard writing grip pattern by the school teachers in our education system. That is the reason all the recruited children could use the force acquisition pen (FAP) to write during the assigned tasks. According to our design rationale (to fit the size and weight of a regular pen) for the FAP, only three sensors could be installed on the pen barrel, so only a tripod pen grasp was allowed in our handwriting testing. The other reason is that all four grip types for the HD group among our recruited children were the so-called mature grip type according to the previous classifications (shown in the following lists). Since all the participants in the HD group had already become used to the mature grip type in their writing performance, the authors thus decided not to specifically mention the details of the grip patterns in the text. However, because our original statement was not clear, the authors added one sentence ”**Although all the children had already been taught to use the dynamic tripod grasp to write in school, the most preferred and frequent manner in which they gripped the pencil was recorded.**” to clarify the method for defining the grip types in this study. The following table also shows the distribution of these categories of handwriting grip in the three types of HD among the recruited children.

In addition, in the design of the FAP, we had to constrain the pen-grip pattern to the tripod grip type for everyone, which may have limited a full understanding of grip kinetics among different pen grip patterns. Therefore, the authors also added this issue as one of the study limitations in the Limitations and Future Studies part as “**Further, a force acquisition pen that is capable for detecting grip kinetics from different pen grip patterns other than the tripod grasp is still needed.**”.

Please refer to Page 7 lines 128-129 and Page 21 lines 375-376.

| **HD subtype** | **Grip pattern** | **Numbers of subjects (%)** |
| --- | --- | --- |
| Cognitive learning dysfunction | Dynamic tripod | 4 (57.1) |
|  | Lateral tripod | 2 (28.6) |
|  | Quadruped | 1 (14.3) |
|  | **Total** | **7 (100)** |
| Motor impairment | Lateral tripod | 6 (75) |
|  | Lateral quadruped | 2 (25) |
|  | **Total** | **8 (100)** |
| Severe hybrid | Dynamic tripod | 1 (7.7) |
|  | Lateral tripod | 6 (46.2) |
|  | Quadruped | 2 (15.4) |
|  | Lateral quadruped | 4 (30.8) |
|  | **Total** | **13 (100)** |

Mature grips citations:

- Koziatek, S. M., & Powell, N. J. (2003). Pencil grips, legibility, and speed of fourth-graders’ writing in cursive. American Journal of Occupational Therapy, 57(3), 284-288.
- Tseng, M. H. (1998). Development of pencil grip position in preschool children. The Occupational Therapy Journal of Research, 18(4), 207-224.

5. There is an important phrase related to this topic whose wording is ambiguous in my opinion:
   “Although the positions of the three force sensors on FAP are adjustable, in this study, all participants used the same position setting for the force sensors based on their own grip.” What does it mean? That all children used the same position setting of the sensors in the pen, regardless of how each child grasped it? Or that each child had an individualized configuration of sensors according to their form of grasping? Or, yet, that there were several configurations, one for each form of handwriting grasping, but not one for each child according to their natural positioning?
   This questioning is important because it can indicate whether there was an extra constraint factor for the HD group. If the positioning was unique, its effects on the HD group may have been greater than on the group without HD. If the positioning was determined by the way of grasping, how many subgroups were formed? And if the positioning was individualized by a child, what did you mean by "same position setting"? Therefore, I believe that, by not adequately considering the heterogeneity of the HD sample, the authors compromised the reliability of the results. As stated by them, “due to the small sample size and uneven percentages among the subtypes, differences between HD subtypes were not analyzed”.
   **Response:** Thanks for the comments. The sentence “Although the positions of the three force sensors on FAP are adjustable, in this study, all participants used the same position setting for the force sensors based on their own grip.” in our original text may have been too vague. To improve readability, the authors have rephrased the statement as follows: **“Although the positions of the three force sensors on the FAP were adjustable,** **no participant in this study, however, requested the examiner to adjust the position of the sensors due to discomfort or an awkward hand posture.”** in the Procedures part of this revised version. Please refer to Page 9 lines 181-182, and Page 10 line 183.

Again, the authors agree with the reviewer’s viewpoint regarding the heterogeneity of the HD participants. Unfortunately, as in our previous response, the objective of this was to reveal the differences in pen grip kinetics in handwriting performance for the HD group and control group. Although distinguishing the differences in the writing kinetics among the HD subtypes is also a critical issue to be investigated, this was not the focus of the present study. Therefore, the authors stated this issue in the Discussion section and the Limitations and Future Studies part of this revised version as follows: **“However, the data in this study were analyzed in children with uneven percentages of distribution among all three subtypes of HD. Since HD is very complex, and an assessment for HD classification has only been developed recently, the results obtained in this study should be interpreted with caution when generalizing the findings to children with different types of HD.”** and **“ Finally, although children with three subtypes of HD were reported according to the CHEF classification, this study did not analyze the differences of the characteristics of writing kinetics among the different HD subtypes due to the small sample size and uneven percentages of distribution among the subtypes in the current study sample. A future study with a larger sampling scale is suggested to compare the differences of the writing features among HD subtypes.”** Please refer to Page 16 lines 262-266 and Page 21 lines 378-383, respectively.

6. Some minor considerations:
   The model of direct citations used in the article (citing only the first two authors) is not adequate, since it gives a single comma the power to indicate that there are other authors besides the two mentioned. Forms such as "Author and colleagues", "Author and collaborators" and even "Author et al." are preferable in these cases.
   **Response:** Thanks for the reviewer’s correction. The authors have revised the description as follows: **“However, Chau and colleagues [15] showed different results in their work regarding pen-grip activity.”**, **“Lin et al. [13] enrolled 181 children ranging in age from 5 to 12 years old and found that the younger children exhibited more force variations and lower frequency of adjustment in the amount of force applied from each digit.”**, **“Regarding the grip-to-tip ratio, which is the total force applied from the grip divided by the force applied from the pen-tip, Lin et al. [13] found that older children tend to apply force on the pen barrel rather than pushing the pen-tip downward. Similarly, Chau et al. [15] found that compared to children with CP, children with typical development generate higher grip-to-tip ratios.”**, **“Ghali et al. [22] suggested that each person has a specific, recognizable force distribution on the pen barrel while writing. Shim et al. [23] also proposed a system measuring contact forces from three digits.”**, **“Drotár et al. [35] suggested that in-air movement during handwriting may involve cognitive processes such as motor planning and programming of motor sequences.”**, and **“Rosenblum et al. [16] found that children with poor handwriting had a lower in-air to total time ratio and a lower speed.”**.

Please refer to Page 4 line 66-67; Page 5 lines 78-80; Page 5 lines 83-87; Page 5 lines 89-91; Page 12 lines 202-203; Page 17 lines 282-283.

7. Although it could be considered as implied, the text does not formally indicate whether the sample is composed only by right-handed children and does not comment on the consistency of manuality. They belong to a key age range regarding manuality definition, and the topic deserves to be considered in the characterization of the sample (Scharoun & Bryden, 2014). Scharoun, S. M. and Bryden, P. J. Hand preference, performance abilities, and hand selection in children. Front Psychol. 2014; 5: 82. doi: 10.3389/fpsyg.2014.00082

**Response:** Thanks for the reviewer’s comment and suggestion. There were three left-handed subjects in the HD group. The authors revised the description as follows: “**Thirty-six children without HD (Control group, 18 girls and 18 boys, mean age 7.97±0.57 years, all right-handed) …3 left-handed and 25 right-handed)… therapy practitioners.**”.

Please refer to Page 8 line 111-113 and Page 9 line 114-116.

8. The Cronbach’s alpha value for the BOT-2 test is 0.92; however, ideal values for Cronbach’s alpha range from 0.7 to 0.9. Values greater than 0.9 indicate test redundancy (Streiner, 2003). Streiner, D. L. Being inconsistent about consistency: when coefficient alpha does and doesn’t matter. Journal of Personality Assessment. 2003; 80:217-222. doi: 10.1207/S15327752JPA8003_01
   **Response:** Thanks for the comments. The authors agree with the reviewer’s viewpoint. There are two fine motor skill assessment tools commonly used in hospitals for evaluating children’s fine motor capability in Taiwan. One is the BOT-2, and the other one is the Movement ABC (Movement Assessment Battery for Children). Both tests are widely used in scientific studies. The BOT-2 uses the same tests for age from 4 to 21 years old. However, the content of the tests in the Movement ABC should be changed depending on the age of the subjects. In order to make further comparisons with other similar studies in the field convenient, the BOT-2 was chosen for our experiment. Although the Cronbach’s alpha value for the BOT-2 test indicates test redundancy, it seems to be one of the best choices to provide an objective assessment of children’s fine motor functions in a clinical setting, as compared to the Movement ABC.

- Croce, R. V., Horvat, M., & McCarthy, E. (2001). Reliability and concurrent validity of the movement assessment battery for children. *Perceptual and motor skills*, *93*(1), 275-280.

9. Right following the phrase “There have been few studies reporting on applied force while pen-lifting” there are no references to these few studies, nor a discussion comparing the results obtained in the study with this literature, which is very relevant here.

**Response:** Thanks for the reviewer’s correction. The authors revised the statements as follows: **“To the best of our knowledge, there are no studies in the literature on applied digit force during pen-lifting. This study may thus be the first to report these data based on the handwriting performance of children.”**.
Please refer to Page 17 lines 276 to 278.

Reviewer #2:

The aim of this study was to characterise the pen-grip kinetics profile of children with and without handwriting difficulties (HD), an issue that has received little attention to date. Despite the relevance of the topic, certain aspects limit the impact of the study, for example, the absence of an in-depth analysis of past studies; the use of a heterogeneous sample of children with handwriting difficulties; and the statistical approach to data analysis. The points that need to be clarified and strengthened are detailed below.
We sincerely appreciate the Reviewer’s thorough review and valuable suggestions. The authors revised the manuscript according to your constructive comments. The point-by-point responses are shown as follows:

Introduction
1. The introduction requires a more detailed review of previous studies. According to the authors, “the results of previous studies related to children’s handwriting kinetics remained inconsistent” (p.7, lines 2-3), meaning that one of the main motivations of the present study was to provide more evidence in regard to the issue. According to the above, the reader would expect to be provided with possible explanations for the heterogeneity of previous results.

However, only in the case of findings concerning variations in terms of grip force were certain methodological reasons mentioned as a source of inconsistency (p.6, lines 13-15). A more in-depth analysis of previous findings would make for a more compelling argument as to the specific contribution of the present study.
**Response:** Thanks for the reviewer’s constructive comments and corrections. The authors agree with the reviewer’s viewpoints regarding the flaws of the description about “the inconsistent issue” in our original Introduction section. In order to concentrate on the main focus of this study, which was an attempt to present “the lack of previous works regarding comprehension of digit forces acting on a pen barrel during handwriting” and also avoid misleading the readers as to the themes addressed in this study, the authors removed the irrelevant statement “**the results of previous studies related to children’s handwriting kinetics remain inconsistent, and**…” in the Introduction section of the revised version of the paper. According to the context of this paragraph, we rephrased the sentence as **“Since studies on digit force are still limited, this study was designed as an observational study to provide insight into the issue.”** so that inconsistencies in the handwriting kinetics (especially those related to the pen-tip force) will not be the main argument in this study. Please refer to Page 6 lines 93-94.

In addition, the authors have searched and added more proper published works as the references in the revised Introduction section: Please refer to Page 6 line 93. While updating this section, the authors also found that research works related to children’s handwriting kinetics have become scarce in recent years. Besides those classic articles related to handwriting kinetics published several decades ago, most of the studies were the works around 2005 to 2015 which were the fabulous time period for the handwriting researchers to discussed the pen tip force based on the mature tablet (Wacom) system. However, the comprehension of the direct evidence regarding the finger/digit forces acting on the pen barrel was still scant so that our team (and other previous works which had been cited in this manuscript) had established a digit forces acquisition pen that was similar to the real pen (either shape or weight/size). With the technology of conducting small force or pressure sensors as well as the 3D printing technical advancements, we believe that handwriting kinetics-related studies might be conducted easily for further discussing the mechanisms of handwriting motor or processing functions. Thanks again for the reviewer’s suggestions and corrections which motived us to update our citations and improve the readability of the revised version of the paper.

2. The research questions overlap to a certain degree. It would be advisable to reformulate them, as this would also help to improve the statistical analysis strategy.
**Response:** Thanks for the reviewer’s constructive suggestions. The authors revised the statements of research questions as **“(1) Are there any differences in the fine motor skills and pen-grip kinetics between children with HD and without HD, and (2) are there any role differences among the thumb, index, and middle finger?”**. Please refer to Page 6 lines 99-101. In addition, the statistical analyses used for examining the questions were also changed to the use of ANCOVAs. The description was revised as **“****To address research question one, an analysis of covariance (ANCOVA) controlling for age was used to analyze the experimental data to determine the between-group differences in the parameters and raw BOT-2 scores. To address research question two, since the participants were compared in two groups, a mixed-model ANCOVA including one between factor (group: with HD vs. without HD) and one within factor (role of the digits: thumb vs. index finger vs. middle finger) was performed on the AF_whole task_, the CVF_whole task_, and the NFFPS _whole task_, with age as a covariate. Bonferroni tests were used as post-hoc tests.”** Please refer to Page 12 lines 214-219; Page13 lines 220-221.

Method
3. My main concerns in this section are in regard to the sample. The group of children classified as having HD is highly heterogeneous according to the profiles extracted from the Chinese Handwriting Evaluation Form (CHEF), so it is difficult to understand the implications of the differences found later with regard to the control group. The difference in the age range between the groups should also be emphasised, as the children with HD had almost an additional year of handwriting practice compared to their non-HD peers, a fact which could explain the lack of differences in some measures.
**Response:** Thanks for the comments. The authors agree with the comment regarding the heterogeneity of children in the HD group. Considering the original and main purpose of this study was to reveal the difference in pen grip kinetics in handwriting performance between an HD group and a control group, the details of the characteristics of the children in HD group were not specifically analyzed. However, since there are very few studies that discuss the digit force in children with HD, the authors believe this study provides new evidence and concept as a pilot study. Again, the authors agree with the reviewer’s concerns. Therefore, the authors stated this issue in the Discussion section and the Limitations and Future Studies part of this revised version as follows: **“However, the data in this study were analyzed in children with uneven percentages of distribution among all three subtypes of HD. Since HD is very complex, and an assessment for HD classification has only been developed recently, the results obtained in this study should be interpreted with caution when generalizing the findings to children with different types of HD.”** and **” Finally, although children with three subtypes of HD were reported according to the CHEF classification, this study did not analyze the differences of the characteristics of writing kinetics among the different HD subtypes due to the small sample size and uneven percentages of distribution among the subtypes in the current study sample. A future study with a larger sampling scale is suggested to compare the differences of the writing features among HD subtypes.”** Please refer to Page 16 lines 262-266 and Page 21 lines 378-383, respectively.

4. The pen-grip patterns were not distributed in the same way between the groups with and without HD. The lateral quadruped grasp was more prevalent within the HD group, while the dynamic tripod grasp was more prevalent within the control group. Do you think this could be a source of explanation for the differences found between the groups? This point is especially relevant if we consider that, as I understand it, the positions of the three force sensors were fixed with a tripod grasp pattern: “Although the positions of the three force sensors on FAP are adjustable, in this study, all participants used the same position setting for the force sensors based on their own grip. They were requested to trace numbers in each trial, with three trials each. They had a one-minute rest interval between each trial and enough time to practice using the FAP with a tripod grasp before the formal trials” (p.10, lines 18-22).
**Response:** Thanks for the comments. The authors agree with the comments that the pen-grip patterns could provide an explanation for the between-group differences. While looking carefully at the distribution of pen-grip patterns between groups, the difference in the dynamic tripod was about 8%, and in the lateral quadpod, it was about 11%. However, considering the small difference and the small sample size, the issue of the distribution of these categories of handwriting grip in the two groups was not discussed. Again, the authors deeply appreciate the reviewer’s constructive comments and understand this issue is worthy of further investigation. The authors thus added the statement regarding the influence of pen-grip type into the study limitations in the Limitations and Future Studies part as “**Further, a force acquisition pen that is capable for detecting grip kinetics from different pen grip patterns other than the tripod grasp is still needed.**” Please refer to Page 21 lines 375-376.

Results
5. This section requires a major revision.
The analysis strategy used to answer the two questions could be improved. To answer research question one (Are there any differences in the fine motor skills and pen-grip kinetics between children with HD and without HD?), a series of t-tests were used to compare the groups using variables that are correlated; this is not a good idea given the increased chance of a type I error. The differences in age should have been controlled.
**Response:** Thanks for the comments and suggestions. Previous studies (see the following lists) usually examined the handwriting performance with different parameters in different periods or under different conditions to determine the specific writing control pattern. Although the variables were correlated, each variable could represent its specific control patterns and writing features. Therefore, the authors used these variables to present their specific control patterns and clinical meanings in Table 1.

Since the age was not controlled well, the authors revised the discussion as follows: **“****The mean age of children with HD was more than 8 months older than the age of the children without HD. This indicated that the children with HD had almost an additional year of handwriting practice compared to their non-HD peers in this study.”** to emphasize the age difference between groups. In addition, as mentioned in the previous question (point 2.), the authors now use ANCOVAs to control for age to address both research questions.

Please refer to Page 7 lines 116-118.

Citations:

- Rosenblum, S., & Livneh-Zirinski, M. (2008). Handwriting process and product characteristics of children diagnosed with developmental coordination disorder. *Human movement science*, *27*(2), 200-214.
- Chang, S. H., & Yu, N. Y. (2013). Handwriting movement analyses comparing first and second graders with normal or dysgraphic characteristics. *Research in developmental disabilities*, *34*(9), 2433-2441.
- Horie, S., & Shibata, K. (2018). Quantitative evaluation of handwriting: factors that affect pen operating skills. *Journal of physical therapy science*, *30*(8), 971-975.

6. To answer research question two (Are there any differences in pen-grip kinetics among different digits that indicate corresponding roles?), three one-way ANOVAs were used for each group. At this point, one wonders why the authors were not interested in the differences between the groups, although this is related to the problem of overlap in the research questions. As the comparison between groups was not of interest to the authors, they compared the force grip of the three digits for each group and parameter. A less complex and more robust approach could have been achieved using a mixed model.
**Response:** Thanks for the comments and suggestions. The authors revised the statements related to the research questions to clarify the study questions of interest. In addition, owing to the clarification of our research questions, the authors also revised the statistical analysis for this second research question from a one-way ANOVA to a mixed-model ANCOVA. This is explained in the paper as follows: **“To address research question one, an analysis of covariance (ANCOVA) controlling for age was used to analyze the experimental data to determine the between-group differences in the parameters and raw BOT-2 scores. To address research question two, since the participants were compared in two groups, a mixed-model ANCOVA including one between factor (group: with HD vs. without HD) and one within factor (role of the digits: thumb vs. index finger vs. middle finger) was performed on the AF_whole task_, the CVF_whole task_, and the NFFPS _whole task_, with age as a covariate. Bonferroni tests were used as post-hoc tests.**” The Results and Discussion sections related to the role differences in the digits have also been revised accordingly.

Please refer to Page 12 lines 214-219 and Page 13 lines 220-221; Page 19 lines 333-345 and Page 20 lines 346-361.

Discussion
7. Given that the main argument of the study was based on the inconsistencies in the findings of previous studies, I would have expected a discussion reporting explicitly on how the results of the present work help to clarify these inconsistencies. However, the discussion does not address this.
The authors appropriately highlight the limitations of the work, but do not address the practical implications that the results could have for the identification and intervention of children with HD.

**Response:** Thanks for the comments and suggestions. As the authors mentioned in their answer to question #1, the main argument in the revised version was changed. The inconsistency in the handwriting kinetics (pen-tip force) is not the main argument in this study. The main focus is more on ‘the lack of studies regarding digit force.’ Therefore, the statement reporting on how the results of the present work might help to clarify these inconsistencies is not explicitly addressed in the Discussion section.

Since the recruited group of HD children could have been heterogeneous in this study, this may lead to it being difficult to directly generalize the findings to all children with HD. This is why the authors have carefully concluded and tried not to overexplain the findings of this study. The implications are mentioned in the Conclusion section as follows: **“On the basis of the results, our findings suggested that when intervening in children’s handwriting problems, it should be noted if the children loosen the gripping digits even when the digits appear to be touching the pen barrel during writing, especially at the moment when they lift the pen to write the next stroke. Digit role differences could also be considered when evaluating writing. Compared to the children with HD, the children without HD used a more static method by which to apply the force required to grip the pen and generated faster force adjustments to manipulate the pen from a kinetic point of view.**”. Please refer to Page 21 lines 386-391 and Page 22 lines 392-394.

Since there are very few studies discussing digit force in children with HD, the authors believe this study provides new evidence and concept as a pilot study. This study was an attempt to explore the digit force quantitatively in children with HD via the use of a force acquisition pen. Although the limitations, such as the heterogeneity of the sample, applicability of the FAP, and the comparison of grip-types exist, this study still provides evidence on the role of force control among digits and a pen during a writing task. Furthermore, the HD classification is still a critical issue to be further explored. As for the CHEF, which is the only published and purchasable standard tool in the traditional Chinese handwriting system, the limitations of this tool, such as the small number of published studies regarding its use or the fact that it is a questionnaire rather a pencil paper test, indicate a need for more research evidence and in-depth discussion. Once the relationship between digits and a pen are revealed for a specific handwriting style or for a specific handwriting problem, standard principles for digit force control patterns can be constructed. Therefore, this study has shown the difference in the digit kinetics between children with and without HD. In future studies, the development of an understanding of different HD subtypes as well as grip patterns during handwriting tasks should be explored.
