# Supplementary material for: Pen-grip kinetics in children with and without handwriting difficulties
Source: PLoS One. 2022 Jun 24;17(6):e0270466. doi: 10.1371/journal.pone.0270466 (PMC9231762; doi:10.1371/journal.pone.0270466)
Supplement: Attachment — Submitted filename: Response to Reviewers.docx [file pone.0270466.s004.docx]

Reviewer #1:

The authors did a good job of reviewing the proposed article following the 1st round of revision. The changes regarding the bibliography used and the statistical analysis raised the quality of the work and the importance of the results presented here.
Since the control group had 25 children, I believe the HD group would be a little less heterogeneous if the three left-handed children were removed from the sample and from the statistics.

That leaves two groups of 25 children, all of them right-handed. However, since the data set are provided with the article, I do not believe that the presence of these children in the sample invalidates the results obtained. It is up to the authors and the editor to assess whether this change is necessary.
The discussion benefited from the changes made to the statistics. However, the three articles added to the Introduction could certainly have been taken up in the discussion, since they are recent and relevant to the discussion of this work.

**Response:** We sincerely appreciate the Reviewer’s thorough review and valuable suggestions. The authors have done a sensitivity analysis by removing three left-handed children. The result is similar to the original data without removing left-handed children. In addition, we have consulted the Biostatistics Consulting Center, Clinical Medicine Research Center, National Cheng Kung University Hospital for the rationality and applicability of the statistics used in this study. For the statistics results after removing three left-handed children, please refer to the following Table A and Table B.

Table A. ANCOVA summary table for determining the effect of the digit, group, and their interaction— removed 3 left-handed children

| Kinetics of handwriting | Covariance  (Age) | | |  | Group | | |  | Digit | | |  | Group 🞨 Digit | | |  |
| --- | --- | --- | --- | --- | --- | --- | --- | --- | --- | --- | --- | --- | --- | --- | --- | --- |
|  | *F* | *Sig.* | *η_p_^2^* |  | *F* | *Sig.* | *η_p_^2^* |  | *F* | *Sig.* | *η_p_^2^* |  | *F* | *Sig.* | *η_p_^2^* | Post-hoc |
| AF_on paper_ | 0.55 | .46 | .01 |  | 4.87 | .03^*^ | .08 |  | 2.57 | .08 | .04 |  | 0.44 | .65 | .01 | Ct>HD |
| AF_in air_ | 0.53 | .47 | .01 |  | 12.74 | .00^**^ | .18 |  | 1.63 | .20 | .03 |  | 1.08 | .34 | .02 | Ct>HD |
| AF_whole task_ | 0.69 | .41 | .01 |  | 5.94 | .02^*^ | .09 |  | 2.18 | .12 | .04 |  | 0.91 | .40 | .02 | Ct>HD |
| CVF_whole task_ | 2.02 | .16 | .03 |  | 6.75 | .01 | .10 |  | 1.36 | .26 | .02 |  | 8.94 | .00^**^ | .13 | Interaction^a^ |
| NFFPS_whole task_ | 0.27 | .61 | .01 |  | 5.73 | .02^*^ | .09 |  | 1.60 | .21 | .03 |  | 1.46 | .24 | .03 | Ct>HD |
| *Note.* AF, average force; CVF, coefficient of variation in force; NFFPS, number of force fluctuations per second; Ct, control group; HD, handwriting difficulty group. Post-hoc type: Bonferroni, ^*^*p* < 0.05 ,^**^*p* < 0.01  ^a^ Simple main effect:  ANCOVA for group difference between digitse(three levles):  level 1[Thumb], *F*=1.06, *p*=.31, *η_p_^2^* =.018; level 2[Index], *F*=20.73, *p*=.00, *η_p_^2^* =.263; level 3[Middle], *F*=.09, *p*=.77, *η_p_^2^* =.001;  One-way ANOVA for digit type difference between groups:  [HD], *F*=8.18*, p* =.00, *η_p_^2^* =.254; Post-hoc: Thumb vs Index, *p* =.01; Thumb vs Middle, *p* =1.00; Index vs Middle, *p* =.02  [Ct], *F*=5.29, *p* =.02, *η_p_^2^* =.131; Post-hoc: Thumb vs Index, *p* =.02; Thumb vs Middle, *p* =0.43; Index vs Middle, *p* =.03 | | | | | | | | | | | | | | | | |

Table B. Descriptive statistics parameters in two groups and the ANCOVA result for between group difference after removing 3 left-handed children

| Kinetics of handwriting  (sources of force) | Group | | | |  |  |
| --- | --- | --- | --- | --- | --- | --- |
|  | Control (n=36) | | HD (n=25) | |  |  |
|  | *Mean* | *SD* | *Mean* | *SD* | *Sig.^a^* | *η_p_^2 a^* |
| Fine motor skills | | | | | |  |
| Fine motor precision | 38.44 | 2.16 | 33.20 | 5.13 | .000^**^ | .484 |
| Fine motor integration | 37.22 | 2.04 | 33.96 | 5.74 | .000^**^ | .237 |
| Manual dexterity | 25.94 | 2.91 | 25.80 | 5.19 | .141 | .037 |
|  |  |  |  |  |  |  |
| Average Force (AF), unit: Newton | | | | | |  |
| Thumb -on paper | 3.07 | 1.31 | 2.62 | 0.61 |  |  |
| Thumb -in air | 1.56 | 0.87 | 1.08 | 0.30 |  |  |
| Thumb -whole task | 2.70 | 1.22 | 2.21 | 0.53 |  |  |
|  |  |  |  |  |  |  |
| Index -on paper | 2.11 | 0.77 | 1.68 | 0.62 |  |  |
| Index -in air | 1.15 | 0.55 | 0.63 | 0.22 |  |  |
| Index -whole task | 1.87 | 0.72 | 1.42 | 0.47 |  |  |
|  |  |  |  |  |  |  |
| Middle -on paper | 1.34 | 0.75 | 1.13 | 0.57 |  |  |
| Middle -in air | 1.05 | 0.49 | 0.77 | 0.32 |  |  |
| Middle -whole task | 1.29 | 0.69 | 1.11 | 0.54 |  |  |
|  |  |  |  |  |  |  |
| Pen-Tip -on paper | 0.95 | 0.29 | 0.75 | 0.33 | .012^*^ | .105 |
|  |  |  |  |  |  |  |
| Task Time (TT) | | | | | |  |
| TT -on paper (ratio) | 0.71 | 0.08 | 0.72 | 0.05 | .672 | .003 |
| TT -in air (ratio) | 0.29 | 0.08 | 0.28 | 0.05 | .708 | .002 |
| TT -whole task (s) | 23.46 | 7.49 | 27.29 | 10.61 | .046^*^ | .067 |
|  |  |  |  |  |  |  |
| Coefficient of Variation in Force (CVF) | | | | | |  |
| Thumb -whole task | 0.38 | 0.09 | 0.40 | 0.08 |  |  |
| Index -whole task | 0.35 | 0.08 | 0.49 | 0.15 |  |  |
| Middle -whole task | 0.42 | 0.14 | 0.41 | 0.12 |  |  |
| Pen-Tip -on paper | 0.36 | 0.05 | 0.34 | 0.07 | .245 | .023 |
|  |  |  |  |  |  |  |
| Number of Force Fluctuations per second (NFFPS) | | | | | |  |
| Thumb -whole task | 1.34 | 0.24 | 1.25 | 0.17 |  |  |
| Index -whole task | 1.36 | 0.17 | 1.26 | 0.23 |  |  |
| Middle -whole task | 1.59 | 0.38 | 1.39 | 0.27 |  |  |
|  |  |  |  |  |  |  |
| Force Ratio (FR) -on paper | 0.16 | 0.05 | 0.10 | 0.04 | .000^**^ | .229 |
| *Note.* SD = standard deviation; HD = handwriting difficulty; ^*^*p* < 0.05,^**^*p* < 0.01  ^a^ Results of the ANCOVA correcting for age as a covariate | | | | | | |

Reviewer #2:

The authors have adequately addressed earlier comments about the introduction section. They have also adjusted the research questions and included “age” as a covariate in the analyses, as suggested. However, limitations remain that have not been properly addressed.
**Response:** We sincerely appreciate the Reviewer’s thorough review and valuable suggestions. The authors revised the manuscript according to your constructive comments. In addition, we have consulted the Biostatistics Consulting Center, Clinical Medicine Research Center, National Cheng Kung University Hospital for the rationality and applicability of the statistics used in this study. The point-by-point responses are shown as follows:

1. Results section
   With regard to the split-plot analysis for AF, why was it used for the “whole task” condition but not for the “in air” and “on paper” conditions?

**Response:** Thanks for the Reviewer’s constructive comments and correction.

The authors overlooked the analysis for AF for “in air” and “on paper” conditions for research question 2. Since the condition may represent different control or writing patterns during handwriting, it should be analyzed in both research questions. Thanks to the Reviewer’s correction again. The authors added these two conditions into the analysis and revised the manuscript and also Table 3. The description was revised as “**A mixed-model ANCOVA including one between factor (group: with HD vs. without HD) and one within factor (role of the digits: thumb vs. index finger vs. middle finger) was performed on the AF_on paper_, the AF_in air_, the AF_whole task_, the CVF_whole task_, and the NFFPS _whole task_, with age as a covariate.”**; **“The results of the mixed-model ANCOVA showed significant differences in the AF_on paper_, the AF_in air_, and the AF_whole task_ of the three digits. All these AFs were significantly lower in the HD group.**”; “**Table 3 shows the results for the effects of group and digit type on the AF_in air_, AF_on paper_, AF_whole task_, CVF_whole task_, and NFFPS_whole task_. There was no interaction effect between the group and digit type when analyzing the AF_in air_, AF_on paper_, AF_whole task_ and NFFPS_whole task_ via the mixed-model ANCOVA controlling for age. No significant difference among digit types was found on the AF_in air_, AF_on paper_, and AF_whole task_ or NFFPS_whole task_. The scores for the AF_in air_, AF_on paper_, AF_whole task_ and NFFPS_whole task_ were found to be significantly greater in the control group.**”.

Please refer to Page 12 lines 215-220; Page 13 line 221; Page 13 lines 229-231; Page 15 lines 242-247; Page 16 Table 3.

2. If I have understood correctly, the Group x Digit type interaction is explained by dividing the interaction into two main effects: “Therefore, the simple main effect was then analyzed with ANCOVA (for the group) and One-Way ANOVA (for the digit type)” (lines 244-245). This approach breaks with the logic of an interaction analysis. It needs to be explained by making (orthogonal) contrasts between the levels of the different factors (e.g., differences between HD and control in level 1 [thumb] and 2 [index] vs level 3 [middle]). This may have been done, but the statistical effects associated with the contrasts are not described.

**Response:** Thanks for the Reviewer’s constructive comments and corrections. The authors added the descriptions in the revised manuscript as “**The ANCOVA result showed that only the CVF_whole task_ of the index in the HD group was significantly greater than in the control group (difference between groups in three digit types [Thumb], F=1.13, *p*=.29, *η_p_^2^* =.018; [Index], F=20.30, *p*=.00, *η_p_^2^* =.250; [Middle], F=0.41, *p*=.53, *η_p_^2^* =.007). The One-Way ANOVA result showed that significant differences were found in HD (difference among digits, *F*=6.61*, p* =.00, *η_p_^2^* =.197) and control (difference among digits, *F*=5.29, *p* =.02, *η_p_^2^* =.131) group. The post-hoc tests showed that the CVF_whole task_ of the thumb and middle finger were found to be significantly higher than the CVF_whole task_ of the index finger in the control group (Thumb vs. Index finger, *p* =.02; Thumb vs. Middle finger, *p* =.43; Index finger vs. Middle finger, *p* =.03), and the CVF_whole task_ of the index finger was found to be significantly higher than the CVF_whole task_ of the thumb in the HD group (Thumb vs. Index finger, *p* =.01; Thumb vs. Middle finger, *p* =.43; Index finger vs. Middle finger, *p* =.14).**”. Please refer to Page 15 lines 250-261.

3. No effect size is described in any of the analyses.
   **Response:** Thanks for the Reviewer’s constructive suggestion. The authors added the effect size (η_p_^2^) in the analyses. Please refer to Page 14 Table 2 and Page 15 lines 252-255 and Page 16 Table 3.
4. The analyses generate redundant results. If CVF, NFFPS and AF (whole task condition) are included in the mixed design, they should not be included in the first analysis.
   **Response:** Thanks for the Reviewer’s corrections. The authors revised the parameters used in the two different ANCOVA analyses to avoid generating redundant results. The description was revised as “**An analysis of covariance (ANCOVA) controlling for age was used to analyze the experimental data to determine the between-group differences in the parameters (the AF_on paper_ of the pen-tip, the TTs, the CVF_on paper_ of the pen-tip, and the FR ) and raw BOT-2 scores. A mixed-model** **ANCOVA including one between factor (group: with HD vs. without HD) and one within factor (role of the digits: thumb vs. index finger vs. middle finger) was performed on the AF_on paper_, the AF_in air_, the AF_whole task_, the CVF_whole task_, and the NFFPS _whole task_, with age as a covariate.**”. Table 2 and Table 3 were also revised. Please refer to Page 12 lines 215-220, Page 13 line 221; Page 14 Table 2; Page 16 Table 3.
5. Another two issues remain unclear:
   The first concerns the heterogeneity of the HD group. The authors include this aspect as a limitation, but it would be necessary to show the **descriptive statistics** of the study variables **for each subtype**, perhaps in supplementary material. This would help to explain how the variables work in each subtype and could provide a better understanding of the differences between HD and control.
   **Response:** Thanks for the Reviewer’s constructive comments. The authors have added a summary table to show the descriptive statistics of the study variables for each subtype in the supplementary material.

Please refer to the supporting information, S1 Table. Summary Table of the Parameters in Three Subtypes of HD Group.

6. The second is related to the procedure, specifically, to the fact that the positions of the three force sensors were fixed with a tripod grasp pattern. The authors state: “Although the positions of the three force sensors on the FAP were adjustable, no participant in this study, however, requested the examiner to adjust the position of the sensors due to discomfort or an awkward hand posture” (lines 181-183). However, they do not indicate whether within the task instructions participants were told that if they are uncomfortable, the position can be adjusted.

**Response:** Thanks for the Reviewer’s constructive comments and corrections. Yes, all the participants were told that the position could be adjusted if uncomfortable. However, the original manuscript was not well stated. Therefore, the authors revised the statements as ” **Although the positions of the three force sensors on the FAP were adjustable, no participant in this study, however, requested the examiner to adjust the position of the sensors due to discomfort or an awkward hand posture when the examiners asked them if they needed to adjust the position of the sensors.**” Please refer to Page 9 lines 181-182, and Page 10 lines 183-184.

7. In the discussion section, a number of points need to be addressed in depth. For example, the results concerning AF-in-air and AF-in-paper are not interpreted. What are the implications of the fact that the differences between the groups in AF are found in the “in air” condition but not “on paper”?

**Response:** Thanks for the Reviewer’s constructive comments.

The authors revised the manuscript and added new statements as “**Based on the result, loosening the fingers when lifting the pen might influence children’s writing performance. When the pen is picked up, the constantly applied force from the digits is not necessary due to not making the ink on paper. It may not be necessary to constantly apply the force in the energy-saving perspective. On the other hand, it may reduce the frequency to enlarge the applied force. Therefore, one possibility is that these children could not generate the force constantly. Another possibility is that these children do not know how to constantly apply force. However, more evidence is needed to confirm this hypothesis, such as the muscle endurance test or skill check test.**”, and “**Since handwriting is complex, the reason resulting HD is various. Therefore, this study focused mainly on one concept, the writing kinetics, to reveal the possible and measurable factors related to children with HD. For example, asking children to strengthen or loosen their force applied from a specific digit is a relatively direct way to change the writing pattern. However, in the real world, we could not know if this is necessary or correct to ask them to change the force if we do not know how much force is poor. Therefore, this study provided the real force data such as the AF value, suggested the more critical situation for observing such as pen in air, and proposed the particular digit for observing such as index finger to tentatively bring out the possible and manipulatable factors related to HD. In addition, this study introduced CVF, NFFPS, and FR to explain the force control pattern in children with HD. The study provided a practical method for the teachers and clinicians to assess children’s handwriting problems based on writing kinetics.**”.

Please refer to Page 17 lines 291-298; Page 20 lines 353-364.
