# Supplementary material for: Pen-grip kinetics in children with and without handwriting difficulties
Source: PLoS One. 2022 Jun 24;17(6):e0270466. doi: 10.1371/journal.pone.0270466 (PMC9231762; doi:10.1371/journal.pone.0270466)
Supplement: Attachment — Submitted filename: Response to Reviewers 0503.docx [file pone.0270466.s005.docx]

Reviewer #1:

I believe that the authors did an excellent job of reviewing and readjusting the manuscript. I consider the article complies Plos One's publishing standards.

**Response:** We sincerely appreciate the Reviewer’s thorough review and comment.

Reviewer #2:

The authors have addressed earlier comments concerning the results and discussion sections and the overall quality of the manuscript has increased. However, the writing of these two particular sections needs to be improved through better organization of the information.
**Response:** We sincerely appreciate the Reviewer’s thorough review and valuable suggestions. The authors revised the manuscript according to your constructive comments. In addition, a native English speaker, who is also a professional proofreader, was invited to help with English editing of the revised manuscript. The point-by-point responses are shown as follows:

Results section
1. The authors state: “An analysis of covariance (ANCOVA) controlling for age was used to analyze the experimental data to determine the between-group differences in the parameters (the AF on paper of the pen-tip, the TTs, the CVF on paper of the pen-tip, and the FR) and raw BOT-2 scores. A mixed-model ANCOVA including one between factor (group: with HD vs. without HD) and one within factor (role of the digits: thumb vs. index finger vs. middle finger) was performed on the AF on paper, the AF in air, the AF whole task, the CVF whole task, and the Handwriting difficulties and handwriting kinetics NFFPS whole task, with age as a covariate” (pp 12-13, Lines 215-221).
The reader would therefore expect the findings to be described in the same order: the ANCOVA results and then the mixed-model ANCOVA results. However, the description is not clear because the results of the two analyses are described together in the same paragraph.

**Response:** Thanks for the Reviewer’s constructive comments and correction. The authors have changed the order according to reviewer’s suggestion. The subtitles as “group differences” and “Role differences” were also removed. Please refer to Page 13 line 221 to Page 14 line 248.

The results of the two analyses were described in different paragraph in the revised manuscript. The description was revised as **“Table 2 shows the means and SDs for all parameters and fine motor scores.**

**Table 2 also shows the ANCOVA results on the group differences for the parameters (AF_on paper_ of the pen tip, TTs, CVF_on paper_ of the pen tip and FR) and fine motor scores. For the fine motor skills, scores for ‘fine motor precision’ and ‘fine motor integration’ were significantly lower in the HD group. The AF_on paper_ of the pen tip was significantly lower in the HD group. For the TTs, between-group significance was only found in the TT_whole task_. For the CVF_on paper_ of the pen tip, no significance was found between groups. Finally, the FR_on paper_ was significantly lower in the HD group.”**. Please refer to Page 13 lines 221-228.

2. Regarding the ANCOVA, the results concerning “fine motor skills” and “AF on paper” are described correctly; however, the results concerning “TTs”, “CVF on paper of the pen-tip”, and the “FR” are not mentioned (see p.13, lines 227-230). Instead, these results are mentioned under the description of the mixed-model ANCOVA findings (p. 13, lines 229-235).
**Response:** Thanks for the Reviewer’s comments. The authors have changed the order by mentioning the results of ANCOVA first and then the results of mixed-model ANCOVA according to reviewer’s suggestion. Therefore, the results concerning “TTs”, “CVF _on paper_ of the pen-tip”, and the “FR” were now under the description of the ANVOVA.

The description was revised as **“Table 2 shows the means and SDs for all parameters and fine motor scores.**

**Table 2 also shows the ANCOVA results on the group differences for the parameters (AF_on paper_ of the pen tip, TTs, CVF_on paper_ of the pen tip and FR) and fine motor scores. For the fine motor skills, scores for ‘fine motor precision’ and ‘fine motor integration’ were significantly lower in the HD group. The AF_on paper_ of the pen tip was significantly lower in the HD group. For the TTs, between-group significance was only found in the TT_whole task_. For the CVF_on paper_ of the pen tip, no significance was found between groups. Finally, the FR_on paper_ was significantly lower in the HD group.”**. Please refer to Page 13 lines 221-228.

3. Furthermore, information concerning the mixed-model ANCOVA results is repeated in the “Group differences” and “Role differences” sub-sections.

**Response:** Thanks for the Reviewer’s constructive comments and correction. The authors have changed the order by mentioning the results of ANCOVA first and then the results of mixed-model ANCOVA according to reviewer’s suggestion. The subtitles as “group differences” and “Role differences” were also removed.

Therefore, the information concerning the mixed-model ANCOVA results was not repeated in the “Group differences” and “Role differences” sub-sections anymore. Please refer to Page 13 line 221 to Page 14 line 248.

Discussion section
4. The same organizational problems occur as in the results section. For example, AF-in-air results are addressed on lines 286-300, but suddenly appear again on lines 316-317. Similarly, the interaction Group x Digit on CVF whole task is described on lines 318-321 and appears again on lines 371-373.

**Response:** Thanks for the Reviewer’s constructive comments and correction.

For the AF-in-air results, the description on lines 316-317 has been removed. The description of AF-in-air results have been revised as **“Although the differences were found in all pen positions, only the difference of AF_in air_ in all digits between groups was practically significant because its effect size is large enough to be meaningful in practice. The AF_in air_ in all digits was practically and significantly lower in the HD group.”**, and **“A low CVF represents a static way of grasping a pen in terms of the amount of force. Although not all CVFs showed significant between-group differences, the mean CVF values for the three digits were smaller in the HD group. Our results indicated that the children with HD used a less stable method by which to control the amount of force applied to manipulate the pen during writing.”**. Please refer to Page 17 lines 282-285; Please refer to Page 18 lines 311-315.

For the interaction Group x Digit on CVF whole task, the description on lines 318-321 has been removed. The description was revised as **“A low CVF represents a static way of grasping a pen in terms of the amount of force. Although not all CVFs showed significant between-group differences, the mean CVF values for the three digits were smaller in the HD group. Our results indicated that the children with HD used a less stable method by which to control the amount of force applied to manipulate the pen during writing.”**. Please refer to Page 18 lines 311-315.

5. Lines 287-290 are difficult to follow: “The AF-whole task and AF-on paper results revealed statistically significant but less practical significance (p=0.02 and 0.03; effect size ηp2 =0.09 and 0.07) between-group differences for any of the three digits. However, the AF-in air in all digits was practically and significantly (p=0.00; effect size ηp2 =0.18) lower in the HD group”.

**Response:** Thanks for the Reviewer’s constructive comments. The authors have revised the description and the statistical data were also removed according to the reviewer’s suggestion. The description was revised as **“Although the differences were found in all pen positions, only the difference of AF_in air_ in all digits between groups was practically significant because its effect size is large enough to be meaningful in practice. The AF_in air_ in all digits was practically and significantly lower in the HD group.”**. Please refer to Page 17 lines 282-285.

6. It is suggested that some introductory lines be included in the first paragraph to remind the reader of the objective of the study.

**Response:** Thanks for the Reviewer’s constructive comments. The introductory lines were added in the first paragraph of the Discussion part according to reviewer’s suggestion. The description was revised as **“HD is a wildly discussed issue that has received considerable attention from clinical and educational professionals. Previous studies have revealed valuable kinematics related to handwriting performance. However, the reports on children’s handwriting performance by investigating the force directly applied from the digit are still limited. This study discovered the pen-grip kinetics during writing via the FAP system of children with HD compared with those without HD. In addition, the relationship among three digits during writing was discussed to better understand the digits’ role.”**. Please refer to Page 16 lines 257-263.

7. It is also suggested that the statistical data be removed from the discussion and introduced in the results section instead.
**Response:** Thanks for the Reviewer’s constructive comments. The authors have removed the statistical data according to the reviewer’s suggestion and also revised the related description. The description was revised as **“Although the differences were found in all pen positions, only the difference of AF_in air_ in all digits between groups was practically significant because its effect size is large enough to be meaningful in practice. The AF_in air_ in all digits was practically and significantly lower in the HD group.”**, and **“A low CVF represents a static way of grasping a pen in terms of the amount of force. Although not all CVFs showed significant between-group differences, the mean CVF values for the three digits were smaller in the HD group. Our results indicated that the children with HD used a less stable method by which to control the amount of force applied to manipulate the pen during writing.”**. Please refer to Page 17 lines 282-285; Please refer to Page 18 lines 311-315.

8. There remain a number of unclear sentences and instances where information could be presented more clearly. The manuscript would therefore benefit from a careful review by a native speaker.

**Response:** We appreciate the reviewer’s suggestion. A native English speaker, who is also a professional proofreader, was invited to help with English editing of the revised manuscript.
